# Depletion of Ras Suppressor-1 (RSU-1) promotes cell invasion of breast cancer cells through a compensatory upregulation of a truncated isoform

**DOI:** 10.1038/s41598-019-46575-0

**Published:** 2019-07-11

**Authors:** Vasiliki Gkretsi, Maria Kalli, Christodoulos Efstathiades, Panagiotis Papageorgis, Vassilios Papanikolaou, Lefteris C. Zacharia, Aspasia Tsezou, Evangelos Athanassiou, Triantafyllos Stylianopoulos

**Affiliations:** 1 Cancer Biophysics Laboratory, Department of Mechanical and Manufacturing Engineering, University of Cyprus, Nicosia, Cyprus; 2 Biomedical Sciences Program, Department of Life Sciences, School of Sciences, European University Cyprus, Nicosia, Cyprus; 3 The Center for Risk and Decision Sciences (CERIDES), Department of Computer Sciences, School of Sciences, European University Cyprus, Nicosia, Cyprus; 4 Biological Sciences Program, Department of Life Sciences, School of Sciences, European University Cyprus, Nicosia, Cyprus; 5 Laboratory of Cytogenetics and Molecular Genetics, Faculty of Medicine, University of Thessaly, Larissa, Greece; 6 Department of Life and Health Sciences, University of Nicosia, Nicosia, Cyprus; 7 Department of Biology, Faculty of Medicine, University of Thessaly, Larissa, Greece; 8 Departments of Surgery, University of Thessaly Medical School, Larissa, Greece

**Keywords:** Breast cancer, Metastasis

## Abstract

Extracellular matrix (ECM)-adhesion proteins and actin cytoskeleton are pivotal in cancer cell invasion. Ras Suppressor-1 (RSU-1), a cell-ECM adhesion protein that interacts with PINCH-1, thus being connected to Integrin Linked Kinase (ILK), alpha-parvin (PARVA), and actin cytoskeleton, is up-regulated in metastatic breast cancer (BC) samples. Apart from the originally-identified gene (RSU-1L), an alternatively-spliced isoform (RSU-1-X1) has been reported. We used non-invasive MCF-7 cells, expressing only RSU-1L, and highly invasive MDA-MB-231-LM2 expressing both isoforms and generated stable shRNA-transduced cells lacking RSU-1L, while the truncated RSU-1-X1 isoform was depleted by siRNA-mediated silencing. RSU-1L depletion in MCF-7 cells resulted in complete abrogation of tumor spheroid invasion in three-dimensional collagen gels, whereas it promoted MDA-MB-231-LM2 invasion, through a compensatory upregulation of RSU-1-X1. When RSU-1-X1 was also eliminated, RSU-1L-depletion-induced migration and invasion were drastically reduced being accompanied by reduced urokinase plasminogen activator expression. Protein expression analysis in 23 human BC samples corroborated our findings showing RSU-1L to be upregulated and RSU-1-X1 downregulated in metastatic samples. We demonstrate for the first time, that both RSU-1 isoforms promote invasion *in vitro* while RSU-1L elimination induces RSU-1-X1 upregulation to compensate for the loss. Hence, we propose that both isoforms should be blocked to effectively eliminate metastasis.

## Introduction

Breast cancer (BC) is the most frequent type of cancer in women. When BC is characterized as non-invasive or *in situ*, the prognosis is far better than when it is characterized as metastatic or invasive. In fact, over 90% of cancer mortality is associated with disseminated disease rather than the primary tumor^1–3^. Thus, gaining more knowledge on the molecular mechanism of metastasis and the key players involved might provide new insights on how to inhibit it. Cancer metastasis is a multi-step process where tumor cells first detach from the primary site, degrade surrounding matrix and intravasate into blood or lymphatic vessels. Cancer cells are then attracted by preferred target tissues where they adhere, migrate, and invade through surrounding matrix to finally establish a colony^2^. Integrins and extracellular matrix (ECM)-related adhesion proteins play an important role in all stages of the metastatic process facilitating the communication between cells and the ECM^4^. They act as adaptor proteins being involved in multiple protein-protein interactions^5^, while being also connected directly or indirectly to the actin cytoskeleton. This connection to actin cytoskeleton allows cells to respond better to mechanical or other stimuli by a number of ways including cell shape modulation or alterations in the migration patterns. Importantly, most of them are involved in cancer progression and metastasis as cell-ECM adhesion disruption is one of the first steps in the process^4,6–8^.

## Ras Suppressor-1 (RSU-1)

gene was originally identified to inhibit Ras-dependent oncogenic transformation^9^, and it was later shown to be localized to cell-ECM adhesions through its physical interaction with Particularly Interesting New Cysteine-Histidine protein (PINCH-1) which was proved both in mammalian cells^10^ and in the cells of Drosophila melanogaster^11^ proving that the interaction is phylogenetically conserved. PINCH-1, in turn, participates in the formation of a stable ternary protein complex at cell-ECM adhesions along with ILK and parvins^12^, connecting the ECM with actin cytoskeleton. Interestingly, in Ras-transformed cells the association of RSU-1 protein with the PINCH1-ILK complex is greatly reduced, indicating that RSU-1 may function through an independent pathway in cancer^13^. Although RSU-1 was linked to Ras-dependent oncogenic transformation^9,14^ and was shown to have anti-tumorigenic effects suppressing cancer cell growth^9,15–17^, its expression level and role in cancer has yet to be defined. One study showed that *RSU-1* gene is frequently missing in human hepatocellular carcinoma^18^, while in another study *RSU-1* mRNA expression was found to be significantly up-regulated in metastatic colorectal tumor samples versus healthy controls or primary samples^19^. It has also been shown that *RSU-1* expression was increased in human BC samples compared to the control which consisted of the patient’s own normal adjacent tissue. In fact, *RSU-1* was found to be more dramatically upregulated in metastatic BC samples compared to non-metastatic (*in situ*)^20^. Notably, *RSU-1* was upregulated in aggressive cell lines of BC^20^ and hepatocellular carcinoma^21^. Moreover, meta-analysis of Affymetrix microarray gene expression data from 5143 BC patients showed that although elevated *RSU-1* mRNA expression was not correlated with overall survival, it was correlated with poor prognosis both in terms of distant metastasis-free survival and remission-free survival^22^. These data indicate that RSU-1 may be involved in BC metastasis, although the underlying mechanism is still vague.

**Figure 1:**
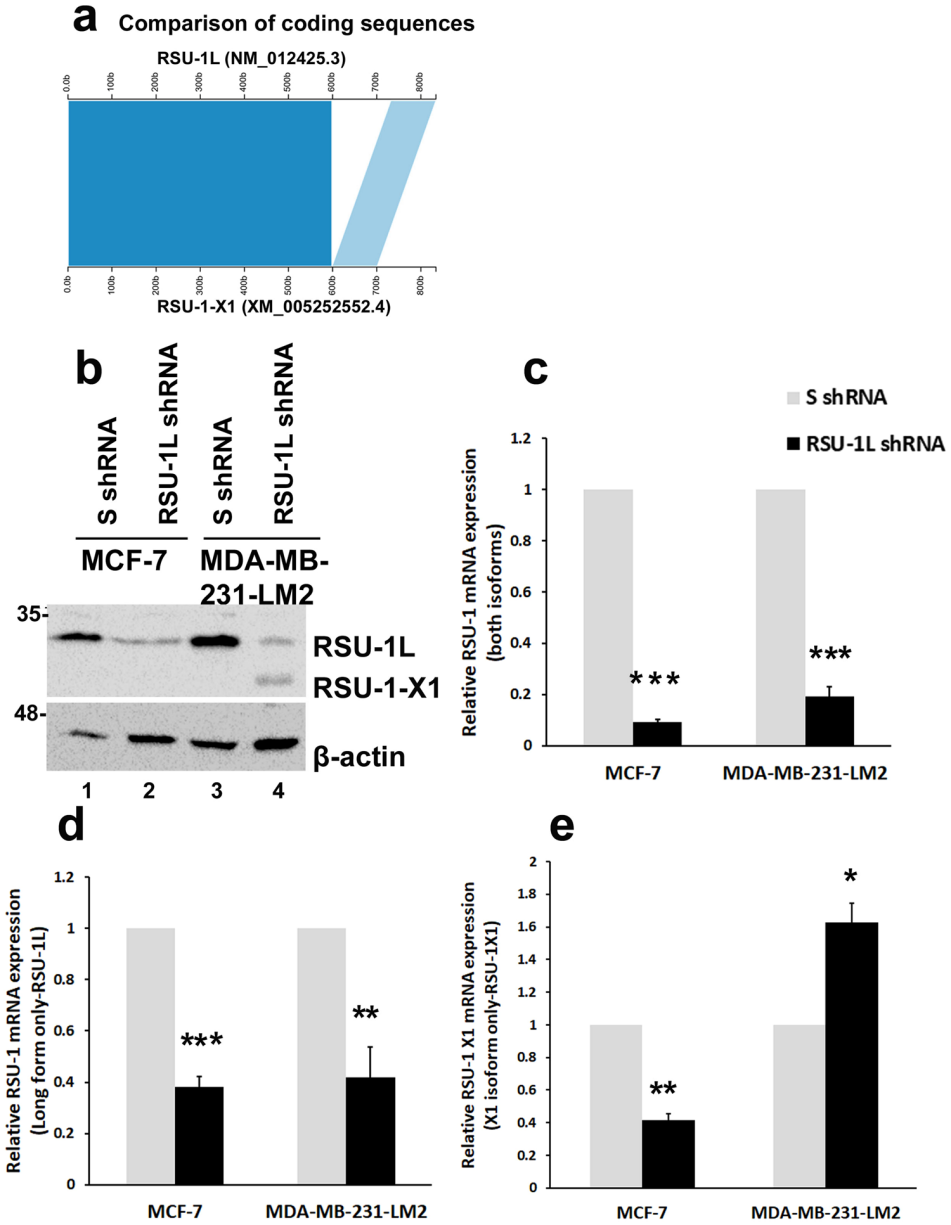
Comparison of the gene sequences of RSU-1L and RSU-1-X1 and effect of RSU-1L depletion from MDA-MB-231-LM2 cells on the expression of the truncated RSU-1-X1 isoform. (**a**) Comparison of the gene sequences of RSU-1L and RSU-1-X1. Trapezoids drawn between the bars indicate the portions of the sequences that align to each other, while the white area corresponds to the missing sequence in RSU-1X1 isoform (nucleotides 598–731, total missing piece of 133 bp). (**b**) Representative western blot using total cell lysates from MCF-7 and MDA-MB-231-LM2 cells stably expressing scrambled control shRNA (SshRNA) or shRNA against RSU-1L (RSU-1L shRNA). Two RSU-1 isoforms are identified while β-actin was used as loading control. (**c**–**e**) Real Time PCR-mediated analysis of mRNA expression using primers that recognize both RSU-1 isoforms (**c**), only RSU-1L (**d**) or only RSU-1-X1 (**e**) isoform. B-actin was used as the housekeeping gene and S-shRNA-treated cells served as calibrator for the ΔΔCt method. Experiments were performed in triplicates and four (4) independent experiments were conducted. Asterisks indicate statistically significant changes (*p-value < 0.05, **p value < 0.01, ***p value < 0.001). Full-length blots are presented in Supplementary Fig. 1.

Interestingly, apart from the originally identified RSU-1 protein of 33KDa (**RSU-1L** herein, NCBI Reference Sequence: NM_012425.3), there is another alternatively-spliced isoform of 29KDa (**RSU-1-X1** herein) reported to be present in more aggressive human gliomas^23^. In fact, there is only one study to date on the role of the truncated RSU-1 isoform^13^, demonstrating that RSU-1-X1 (RSU-1J at the time, but RSU-1 variant X1 according to NCBI, Reference Sequence: XM_005252552.4) does not bind to PINCH-1 and promotes cell migration (See Fig. 1a for comparison of the two isoform sequences).

In the present study, we utilized two BC cell lines of different invasive capacity, namely MCF-7 cells and MDA-MB-231-LM2 cells^24^ to investigate the role of the two *RSU-1* isoforms in BC cell invasion. Our aim was to test if the two isoforms act in concert to exert their action on cell invasion, what is the relationship between them and what happens if one of them is missing. Finally, we validated our findings using human BC samples that expressed differential levels of the two RSU-1 isoforms.

## Results

### Depletion of RSU-1L from MDA-MB-231-LM2 cells leads to upregulation of the truncated RSU-1-X1 isoform

Intrigued by our previous work showing that siRNA-mediated silencing of *RSU-1*, using a commercially available pool of siRNAs that targets both isoforms, leads to reduced invasive capacity of BC cells accompanied by inhibition of urokinase Plasminogen Activator (uPA), and metalloproteinase-13 (MMP-13)^22^, we set out to further investigate the role of *RSU-1* isoforms in BC cell metastasis. First, using a bioinformatics approach, we took advantage of Kablammo, a web-based application that produces interactive, vector-based visualizations of sequence alignments generated by NCBI BLAST in order to compare the sequences of the two *RSU-1* isoforms. As shown in Fig. 1a, RSU-1-X1 lacks a portion of the RSU-1L sequence and specifically nucleotides 598–731 (133 bp). In this figure, the trapezoid drawn between the bars indicates the portions of the sequences that align to each other, while the white area corresponds to the missing sequence.

To study the role of the two isoforms, we decided to silence their expression. However, as siRNA-mediated gene silencing presented several technical constrictions due to its transient nature, and in order to facilitate our study, stable short hairpin-RNA (shRNA)-infected cell lines were generated to continuously express RSU-1L shRNA, thus inhibiting the expression of the long RSU-1 isoform. A scrambled control shRNA (S shRNA) containing a scrambled sequence that does not target any gene was used as our control sample.

We tested the efficiency of the silencing approach by Real Time PCR and immunoblotting in the low-invasiveness MCF-7 cells and the highly invasive MDA-MB-231-LM2 cells. MDA-MB-231-LM2 cells represent a highly metastatic variant of the parental MDA-MB-231 cells, which express both *RSU-1* isoforms^22^ and have been shown to efficiently colonize the lungs when implanted in the mammary fat pad of mice^25^. MCF-7 cells express only one *RSU-1* isoform (*RSU-1L*) as *RSU-1-X1* is only detected by the very sensitive method of Real Time PCR at low levels while MDA-MB-231-LM2 cells also express the truncated *RSU-1-X1* form (Fig. 1b). Using an shRNA-infection approach, *RSU-1L* was indeed effectively silenced in both cell lines both at the protein (Fig. 1b, compare lane 2 to 1 and 4 to 3) and the mRNA level (Fig. 1c,d). Interestingly however, when *RSU-1L* was silenced in MDA-MB-231-LM2 cells, RSU-1-X1 was dramatically upregulated as seen by the appearance of a second shorter band in this sample (Fig. 1b). Increased expression of *RSU-1-X1* at the mRNA level was also observed (Fig. 1e, presumably due to an attempt of the cell to compensate for RSU-1L loss.

### RSU-1L depletion dramatically decreases tumor spheroid invasion in MCF-7 cells but leads to increased invasion of MDA-MB-231-LM2 spheroids

**Figure 2:**
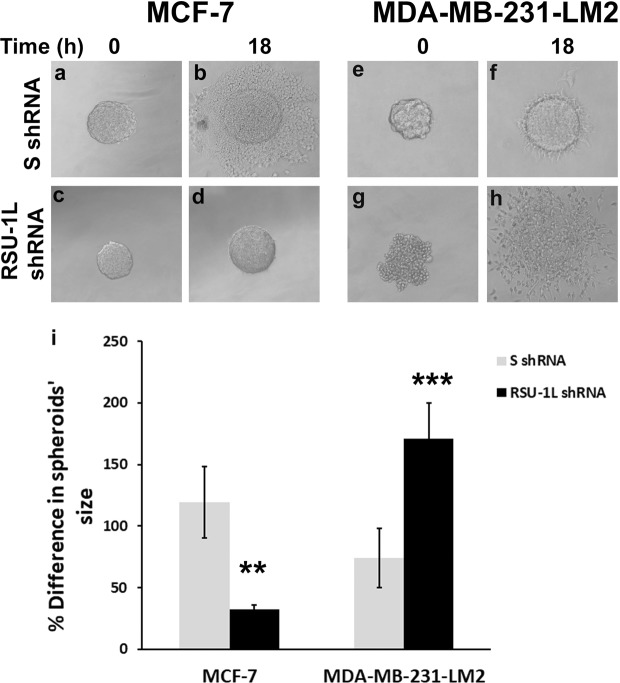
RSU-1L depletion from MCF-7 abolishes tumor spheroid invasion, while its depletion from MDA-MB-231-LM2 enhances invasion. Tumor spheroid invasion assay was performed: (**a–d**) in MCF-7 cells expressing control SshRNA construct (**a**,**b**) or RSU-1L shRNA (**c**,**d**,**e–h**) in MDA-MB-231-LM2 cells stably transfected with control (**e**,**f**) or RSU-1L shRNA (**g**,**h**). (**i**) Percentage (%) change in MCF-7, and MDA-MB-231-LM2 spheroids’ dimensions (average of major and minor axis) within 18 h. At least 8 spheroids were analyzed per condition and 3 independent experiments were performed. Asterisks indicate statistically significant changes (*p value < 0.05, **p value < 0.01, ***p value < 0.001) compared to the condition of SshRNA-treated cells.

Cells were then subjected to tumor spheroid invasion assay in order to test their ability to invade through three dimensional (3D) collagen gels following depletion of RSU-1L. Results were striking, showing that in tumor spheroids formed from MCF-7 cells lacking RSU-1L, their invasion capacity was completely abolished (Fig. 2, compare a-b to c-d, and Fig. 2i) whereas, invasion of MDA-MB-231-LM2 spheroids lacking RSU-1L was dramatically increased (Fig. 2, compare e-f to g-h, and Fig. 2i). Our findings indicate that the two cell lines have differential response to RSU-1L depletion with regard to tumor spheroid invasion.

### Silencing of both RSU-1 isoforms in BC lines

Based on these results, we hypothesized that in highly invasive MDA-MB-231-LM2 cells, depletion of RSU-1L triggers the activation of the truncated RSU-1-X1 isoform to compensate for the loss of RSU-1L function and for this reason its depletion promotes tumor spheroid invasion rather than inhibits it (Fig. 2).

**Figure 3:**
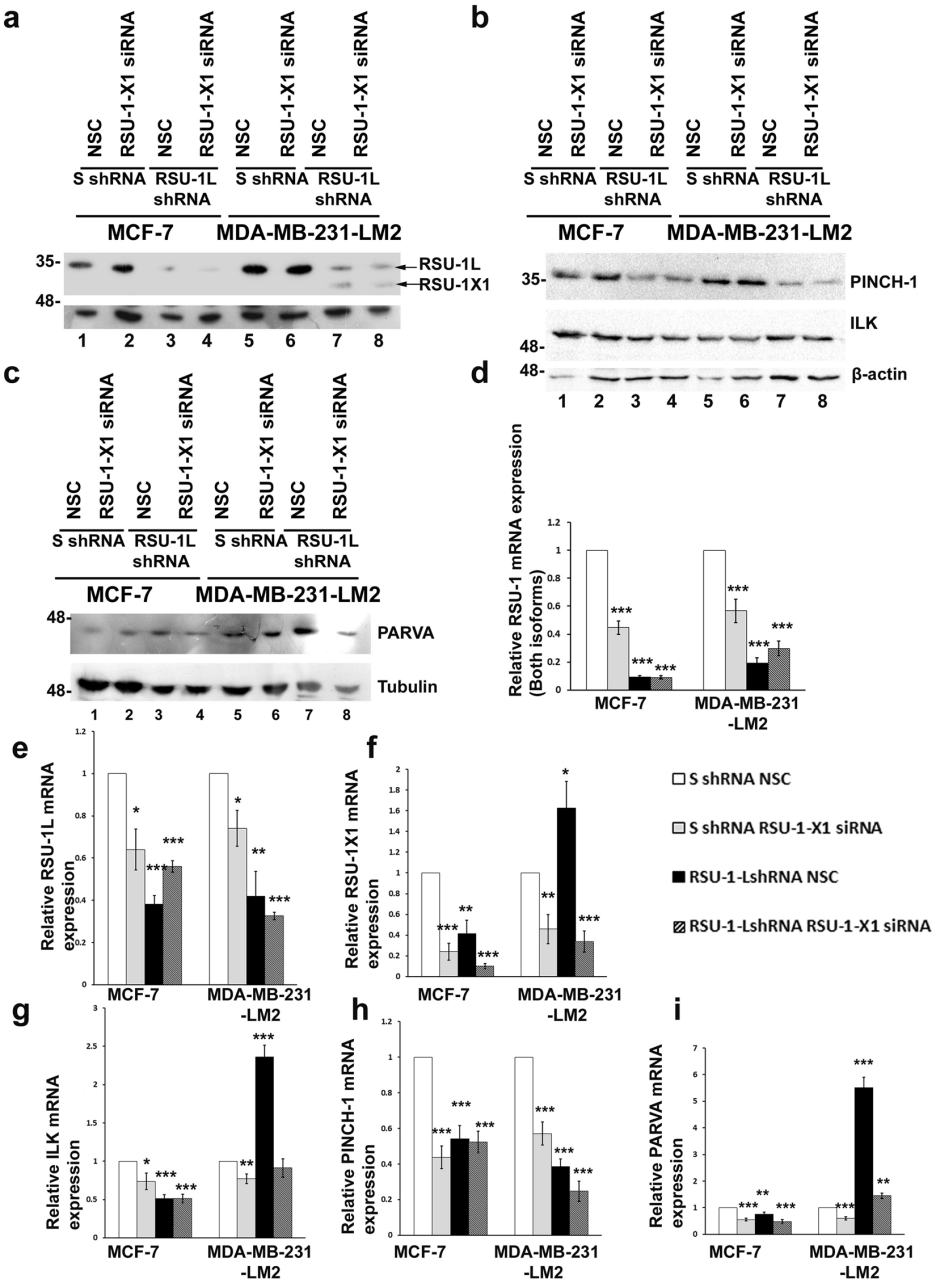
Expression of PINCH-ILK-PARVA upon silencing of one or both of the RSU-1 isoforms. (**a–c**) Protein expression of RSU-1 (**a**), Integrin Linked Kinase (ILK), Particularly Interesting New Cysteine-Histidine rich protein (PINCH) (**b**) and alpha-parvin (PARVA) (**c**) following silencing of one or both of the RSU-1 isoforms. B-actin and tubulin were used as loading controls as indicated. d-f) Real Time PCR analysis of mRNA expression of RSU-1 (both isoforms shown in **d**), RSU-1L (**e**) and RSU-1-X1 (**f**) isoforms. B-actin was used as the housekeeping gene and S-shRNA-treated cells served as calibrator for the ΔΔCt method. Experiments were performed in triplicates and two (2) independent experiments were conducted. Asterisks indicate statistically significant differences (*p-value < 0.05, **p value < 0.01, ***p value < 0.001). (**g–i**)Quantification of the mRNA expression of ILK (**g**), PINCH-1 (**h**) and PARVA (i) under the same conditions using real time PCR. The graphs represent the mean relative mRNA expression of two independent experiments performed in triplicates. An asterisk denotes a statistically significant difference (*p value < 0.05, **p value < 0.01, ***p value < 0.001). Full-length blots are presented in Supplementary Fig. 2 (for **a**), Supplementary Fig. 3 (for **b**) and Supplementary Fig. 4 (for **c**).

To test our hypothesis, we utilized siRNA-mediated silencing targeting the truncated *RSU-1-X1* isoform to transfect the cell lines that stably expressed *RSU-1L* shRNA thus, achieving double knock-down of both RSU-1 isoforms. As shown in Fig. 3, *RSU-1L* was stably silenced in these cells (Fig. 3a, compare lanes 3 and 4 to 1 and 2 for MCF-7, and 7 and 8 to 5 and 6 for MDA-MB-231-LM2 and Fig. 3d,e, solid black and striped bars). When *RSU-1L* was depleted in MDA-MB-231-LM2 cells, *RSU-1-X1* was upregulated (Fig. 3a, lanes 7 and 8 and Fig. 3f, solid black bar). However, upon *RSU-1-X1* siRNA-mediated silencing, *RSU-1-X1* was reduced (Fig. 3a, compare lane 8 to lane 7 and Fig. 3f, solid grey and striped bars compared to the white bar).

### Both RSU-1 isoforms regulate the expression of ILK, PINCH-1 and PARVA

RSU-1L protein is known to bind to PINCH-1 at the cell-ECM adhesion sites, being connected to the ILK-PINCH-PARVA tertiary complex at cell-ECM adhesions^10^, while RSU-1-X1 isoform is postulated to bind poorly or not at all to PINCH-1^13^, which means that it does not actively compete with RSU-1L for PINCH-1 binding and therefore we wouldn’t expect its silencing to have a dramatic effect on PINCH-1 expression. Thus, we set out to test whether silencing one of the RSU-1 isoforms or both would affect the expression of the proteins comprising the PINCH-ILK-PARVA complex in BC cells. MCF-7 cells that express almost exclusively the long RSU-1L isoform offer a more straightforward explanation for the effect of silencing one of the RSU-1 isoforms on PINCH-ILK-PARVA expression. As shown in Fig. 3, RSU-1X1 silencing in both cells lines resulted in downregulation of all three members of the PINCH-ILK-PARVA complex (Fig. 3b,c, compare lanes 1 to 2 for MCF-7 and 5 to 6 for MDA-MB-231-LM2 noting that in 3b lanes 1 and 5 are underloaded as judged by β-actin expression, along with Fig. 3g–i, compare solid gray to white bars). Furthermore, RSU-1L silencing in both cell lines leads to reduced PINCH-1 expression regardless of the presence or absence of the truncated RSU-1X1 isoform (Fig. 3b, compare lanes 3 and 4 to 1 and 2, and lanes 7 and 8 to 5 and 6 for PINCH-1 protein expression, as well as Fig. 3h, solid black and striped bars compared to white for mRNA expression). With regard to ILK and PARVA, RSU-1L silencing reduces their expression in MCF-7 cells (Fig. 3b, compare lane 3 to 1 for ILK and PARVA, as well as Fig. 3g,i, compare solid black bars to white ones) but increases it in MDA-MB-231-LM2 cells (Fig. 3b, compare lane 7 to 5 for ILK and PARVA, as well as Fig. 3g,i, compare solid black bars to white bars). Interestingly, concurrent silencing of the truncated isoform reverses this effect (Fig. 3b, compare lane 8 to 7 for ILK and PARVA, as well as Fig. 3g,i, striped bars compared to solid black bars) indicating that the increase was likely related to upregulation of RSU-1X1 expression.

### Increased migration of highly invasive MDA-MB-231-LM2 cells lacking RSU-1L is significantly reduced upon silencing of the second RSU-1 isoform, RSU-1-X1

**Figure 4:**
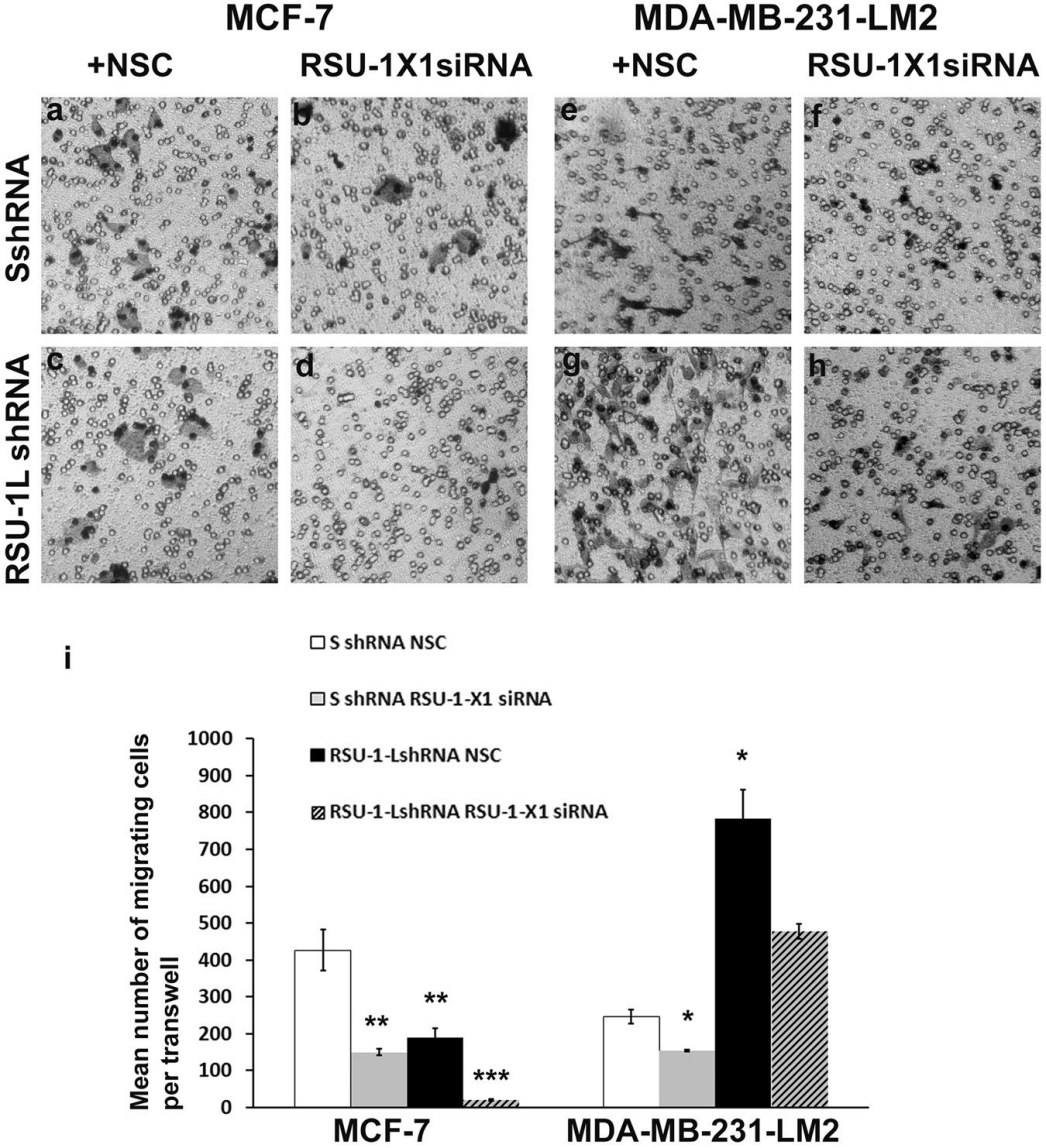
Cell migration in MCF-7 and MDA-MB-231-LM2 cells lacking one or both *RSU-1* isoforms. (**a–h**) Transwell migration assay performed in MCF-7 and MDA-MB-231-LM2 cells transfected with control Non Specific Control (NSC) siRNA and control shRNA (**a**,**e**, respectively), cells transfected with control shRNA and RSU-1-X1 siRNA (**b**,**f**), cells infected with RSU-1LshRNA and transfected with control NSC siRNA (**c**,**g**) and cells transfected with both RSU-1X1 siRNA and RSU-1L-shRNA (**d**,**h**). (**i)** Quantification of the cell migration results from two independent experiments run in duplicates (two transwells per condition). Asterisks denote statistically significant differences (*p value < 0.05, **p value < 0.01, ***p value < 0.001).

Using the experimental set-up described above, MCF-7 and MDA-MB-231-LM2 cells expressing both isoforms (transfected with the Scrambled SshRNA + Non Specific Control-NSC for the siRNA approach), only one (RSU-1L shRNA + NSC or SshRNA + RSU-1-X1siRNA) or none (RSU-1L shRNA + RSU-1-X1 siRNA) of the *RSU-1* isoforms were subjected to transwell migration assay. As shown in Fig. 4, migration of MCF-7 cells was significantly reduced when one of the *RSU-1* isoforms was missing (Fig. 4b,c, compared to 4a) but was dramatically abolished when both isoforms were silenced (Fig. 4d,i). In MDA-MB-231-LM2 cells, depletion of *RSU-1-X1* lead to reduced cell migration (Fig. 4f, compared to 4e), depletion of RSU-1L lead to increased cell migration (Fig. 4g compared to 4e,i), while depletion of both was able to partially reverse the increased cell migration observed due to *RSU-1L* silencing (Fig. 4h compared to 4g,i).

### Increased invasion of RSU-1L-depleted MDA-MB-231-LM2 cells is significantly reduced upon silencing of the second RSU-1 isoform, RSU-1-X1

**Figure 5:**
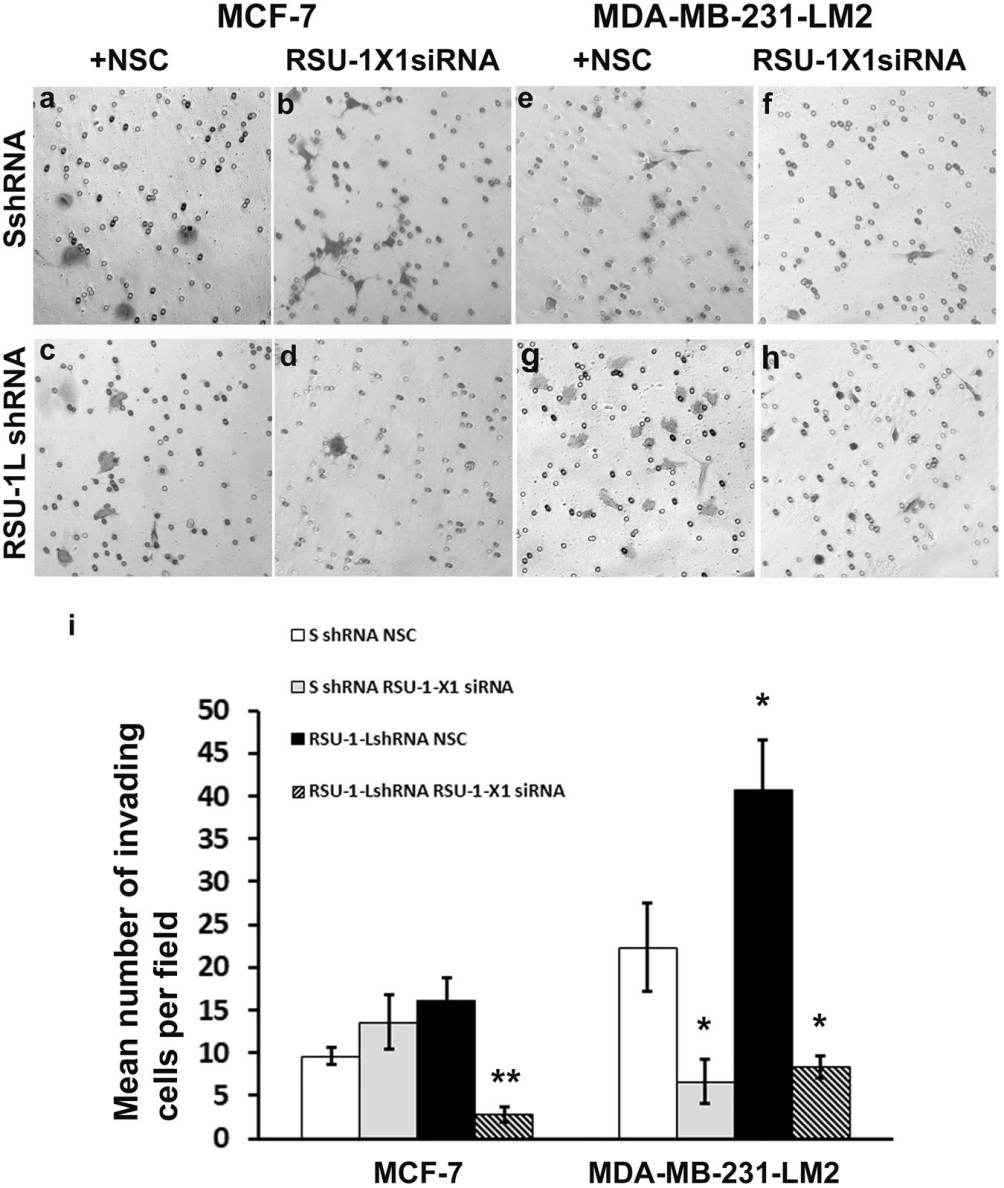
RSU-1-X1 depletion reduces cell invasion, that of RSU-1L enhances it and silencing both decreases it. (**a–h**) Transwell invasion assay performed in MCF-7 and MDA-MB-231-LM2 cells transfected with Non Specific control (NSC) siRNA and control shRNA (**a**,**e**), control shRNA and RSU-1-X1 siRNA (**b**,**f**), RSU-1LshRNA and control NSC siRNA (**c**,**g**) and both RSU-1X1 siRNA and RSU-1L-shRNA (**d**,**h**,**i)** Quantification of the cell invasion results from two independent experiments run in duplicates. Asterisks denote statistical significant differences (*p value < 0.05, **p value < 0.01, ***p value < 0.001).

Similar results were obtained when the traditional transwell invasion assay was performed. MCF-7 cells, which are considered to be low-invasive, showed an effect only when both isoforms were missing. More specifically, as demonstrated in Fig. 5, cell invasion was not dramatically affected in MCF-7 cells lacking RSU-1X1 (Fig. 5b compared to 5a and 5i, solid gray bar) or RSU-1L (Fig. 5c compared to 5a and 5i, solid black bar), perhaps due to the fact that the invasive capacity of these cells is already too low, while depletion of both isoforms completely abolished cell invasion (Fig. 5d compared to 5a–c and 5i, striped bar). In the highly invasive MDA-MB-231-LM2 cells, cell invasion was reduced upon RSU-1X1 silencing (Fig. 5f compared to 5e and 5i, gray bar) and enhanced when only *RSU-1L* was missing (Fig. 5g compared to 5e, and 5i, solid black bar), but was dramatically impaired when both isoforms were silenced (Fig. 5h,i, striped bar), indicating that *RSU-1-X1* inhibition has the potential to reverse the effect of *RSU-1L* silencing on cell invasion.

**Figure 6:**
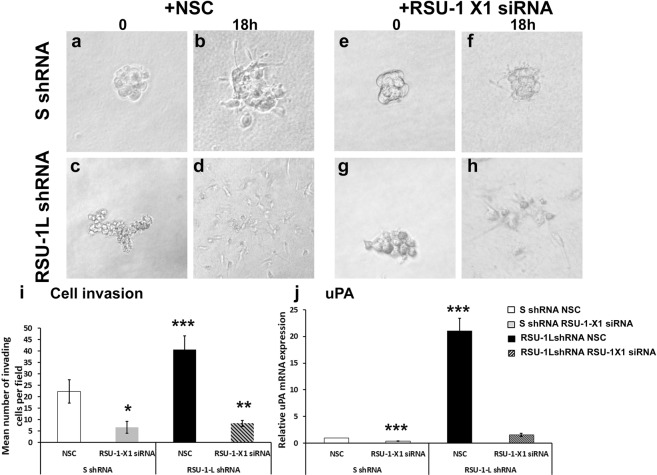
Effect of silencing of one or both of the *RSU-1* isoforms in tumor spheroids invasion. Tumor spheroid invasion assay performed in spheroids from MDA-MB-231-LM2 cells transfected with Non Specific control (NSC) siRNA and control shRNA at zero and 18 h post embedding into collagen gels (**a**,**b**), control shRNA and RSU-1-X1 siRNA (**e**,**f**), RSU-1LshRNA and control NSC siRNA (**c**,**d**) and both RSU-1X1 siRNA and RSU-1L-shRNA (**g**,**h**). (**i**) Quantification of the cell invasion results from two independent experiments with at least 8 spheroids per condition. (**j**) Quantification of the mRNA expression of urokinase Plasminogen Activator (uPA) under the aforementioned conditions by Real Time PCR. Asterisks denote statistical significant differences (*p value < 0.05, **p value < 0.01, ***p value < 0.001).

To verify these results in 3D, we performed a tumor spheroid invasion assay for the same experimental groups in the highly invasive MDA-MB-231-LM2 cell. To do that, stable cell lines lacking *RSU-1L* were transiently transfected with NSC siRNA or RSU-1-X1 siRNA, tumor spheroids were formed and the next day they were embedded in collagen I gels where they were left to invade in 3D. As the transfection period limit for optimal siRNA efficiency is 72 h, spheroids were left to grow for only 24 h, limiting their size (compared to the better formed spheroids in Fig. 2 and affecting their morphology). Nevertheless, the results of the tumor spheroid invasion assay in MDA-MB-231-LM2 cells confirmed the transwell invasion results, demonstrating that depletion of *RSU-1-X1* inhibited spheroid invasion (Fig. 6, compare e and f to a and b, and i, gray bar), and depletion of *RSU-1L* enhanced spheroid invasion (Fig. 6, compare c and d to a and b, and i, solid black bar). Notably, depletion of both isoforms reduced invasion compared to the RSU-1L-depleted spheroids but did not abolish it (Fig. 6, compare g and h to a and b, and i, striped bar). Furthermore, expression of uPA, a protease that has been fundamentally implicated in matrix degradation and cancer cell invasion and metastasis^26^ and it was previously shown to be affected by *RSU-1* silencing^22^, follows the same pattern as spheroid invasion (Fig. 6j).

### RSU-1L and RSU-1-X1 are differentially expressed in metastatic and *in situ* human BC samples with RSU-1L being upregulated in metastatic and RSU-1-X1 being downregulated in the same samples

**Figure 7:**
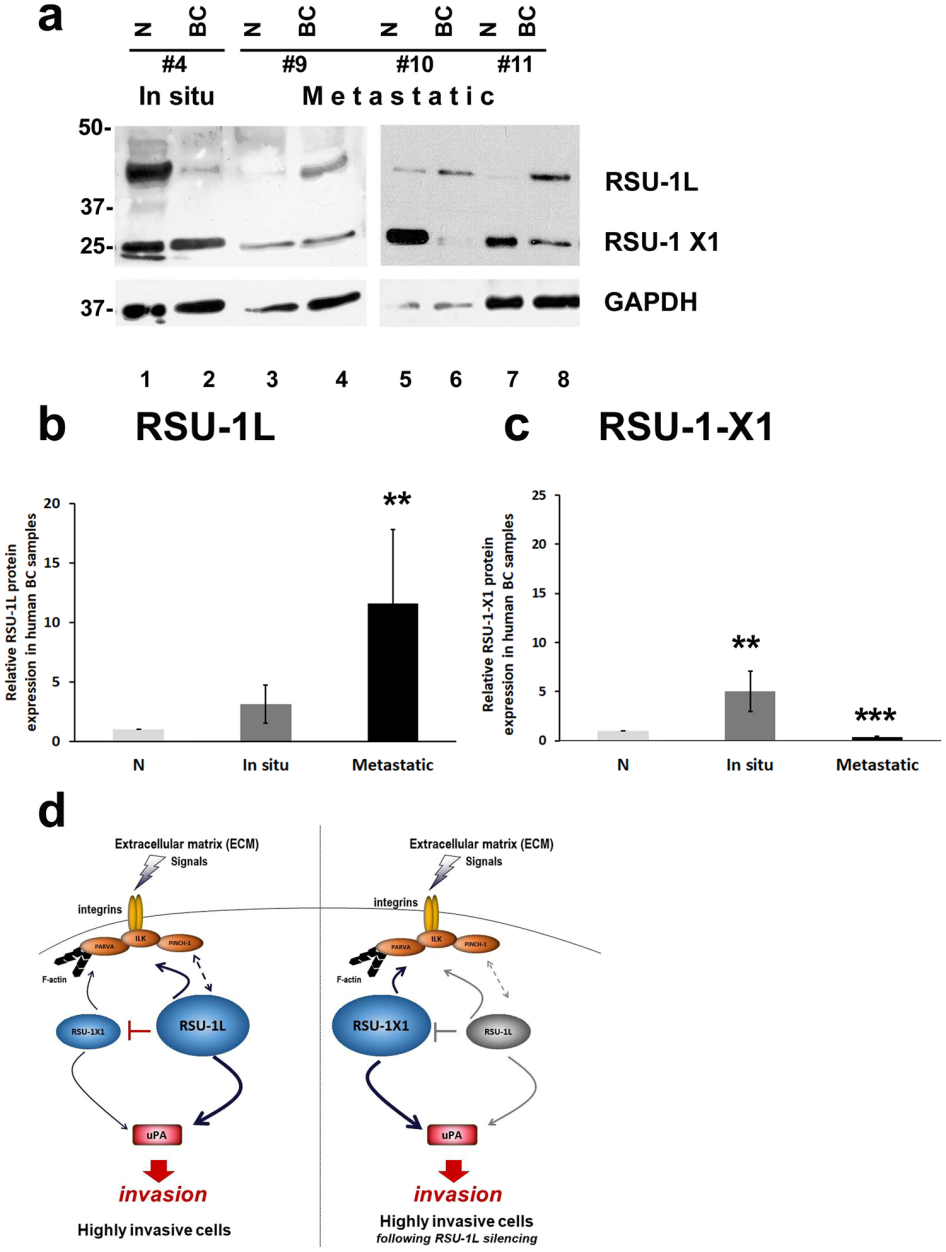
RSU-1 isoforms protein expression in human BC samples from *in situ* or metastatic tumors and diagrammatic overview of the proposed model by which the two RSU-1 isoforms function. Protein expression of RSU-1 isoforms assessed by immunoblotting of total cell lysates from human BC samples taken from patients with *in situ* or metastatic tumors^20^ and their normal adjacent tissue (N). (**a**) Representative immunoblot including one *in situ* and 3 metastatic BC samples. Samples were run on a 15% polyacrylamide gel to be able to also detect the truncated RSU-1-X1 isoform (~29 KDa). Glyceraldehyde-3-Phosphate Dehydrogenase (GAPDH) was used as loading control. (**b**,**c**) Quantification of the band intensity in RSU-1L (**b**) and RSU-1-X1 (**c**) bands from five (5) different immunoblots which included 23 human BC samples. A non parametric Mann Whitney test was used to statistically analyze the data as mentioned in the materials and methods section. An asterisk indicates a statistically significant difference (*p value < 0.05, **p value < 0.01, ***p value < 0.001). Full-length blots are presented in Supplementary Fig. 5. (**d**) Schematic representation of the main findings of this work. Left panel: In highly invasive BC cells, RSU-1L is upregulated while RSU-1-X1 is minimally expressed. Thus, RSU-1L activates PINCH-ILK-PARVA complex as well as urokinase Plasminogen Activator (uPA) leading to invasion. Note that RSU-1L exerts an inhibitory effect on the shorter RSU-1-X1 isoform. Right panel: Upon RSU-1L blocking (e.g. silencing), RSU-1-X1 is dramatically upregulated exerting all the effects that were normally exerted by RSU-1L to compensate for its loss, leading again to invasion.

To better comprehend the role of each one of the *RSU-1* isoforms in BC metastasis, we proceeded to test their expression in total cell lysates isolated from BC patients with *in situ* or metastatic tumors, forming metastasis to at least one lymph node, compared to respective normal adjacent tissue for each tumor. It has been shown that RSU-1L is upregulated in metastatic BC samples compared to *in situ*^20^ but no information was obtained regarding the expression of the RSU-1-X1 isoform. In the present study, we evaluated the expression of both RSU-1 isoforms at the protein level and, as shown in Fig. 7, RSU-1L was dramatically upregulated in metastatic samples (Fig. 7a, compare lanes 4, 6 and 8 to 3, 5 and 7, as well as Fig. 7b), while RSU-1-X1 had the exact opposite expression pattern, being upregulated in *in situ* (Fig. 7a, compare lane 2 to 1, as well as Fig. 7c) and downregulated in metastatic BC samples (Fig. 7a, compare lanes 4, 6 and 8 to 3, 5 and 7 for RSU-1-X1, as well as Fig. 7c).

Information on the patients’ age, family history of BC, height, weight, body-mass-index (BMI), age of menarche and menopause, number of pregnancies, smoking habits, grade, infiltration of the blood vessels, nerves, skin or nipple, pathology evaluation, tumor diameter, status of estrogen receptors (ER), progesterone receptors (PR), HER2/neu, and lymph node metastasis (Supplementary Table 3) was also available. Interestingly, all samples with elevated RSU-1L and reduced RSU-1-X1 were from women with no family history of BC who had had more than one pregnancies and exhibited lymph node metastasis. These findings in human BC samples provide a clinical insight and corroborate our results in the two BC cell lines highlighting the importance of RSU-1 in BC cell metastatic behavior.

## Discussion

Although *RSU-1* was connected to Ras-mediated oncogenic transformation^9,14^, its expression in cancer tissues, and its association to metastasis are still vague. Several reports have demonstrated upregulation of *RSU-1* in some cancer types, such as metastatic colon cancer^19^, metastatic breast cancer^20^, and hepatocellular carcinoma cells^21^. All the above reports, however, refer to the long *RSU-1L* isoform. Regarding the truncated *RSU-1-X1* form, there are only two reports so far; one that comments on its expression in high grade gliomas^23^, and another one reporting its detection in tumor cell lines with high levels of activated *Ras*^13^. Regarding the effect of the RSU-1 isoforms in cancer cell migration, the study by Dougherty *et al*.^13^, showed that RSU-1L inhibits it, perhaps through binding to the PINCH-ILK-PARVA complex, whereas RSU-1-X1 promotes it. In the present study, we defined, for the first time, the connection between the two RSU-1 isoforms. We demonstrated that depletion of *RSU-1L* using an shRNA-mediated approach completely abolishes spheroid invasion in MCF-7 cells (Fig. 2a–d,i) that express almost exclusively this isoform (Fig. 1b) indicating that RSU-1L by itself promotes cell invasion. Similarly, silencing of RSU-1-X1 in MCF-7 cells by an siRNA-mediated approach led to inhibition of cell migration (Fig. 4) also pointing to the direction that RSU-1-X1 by itself promotes metastatic properties. However, it had no significant effect on MCF-7 cell invasion, perhaps due to the fact that MCF-7 are cells of low-invasiveness which means that the detected invading cells were too few to reach statistical significance. It should be noted though that despite the fact that MCF-7 cells do not seem to express RSU-1X1 isoform at the protein level (as evident by immunoblotting analysis, e.g. Figs 1b, 3a), they do have detectable levels of RSU-1X1 mRNA expression as seen by the more sensitive and quantitative method of real time PCR analysis (Figs 1f, 3d), and that is why RSU-1X1 silencing has an effect on cell migration (Fig. 4) and invasion when RSU-1L is also depleted (Fig. 5, striped bar).

In accordance with findings in MCF-7 cells, RSU-1X1 silencing in highly invasive MDA-MB-231-LM2 cells severely impaired cell migration (Fig. 4), and cell invasion (Figs 5, 6). However, depletion of RSU-1L from MDA-MB-231-LM2 cells had opposite effects inducing a dramatic increase in tumor spheroid invasion (Fig. 2e–h,i). Notably, we also noticed a concurrent upregulation of RSU-1-X1 isoform upon *RSU-1L* depletion (Fig. 1b, lane 4 compared to lane 3, and 1e). Thus, we hypothesized that the cell upregulates RSU-1-X1 in an attempt to compensate for RSU-1L loss and that explains the observed increase in spheroid invasion (Fig. 2).

Hence, siRNA-mediated silencing of RSU-1-X1 in cells that already lack RSU-1L showed diminished cell migration (Fig. 4), and cell invasion (Figs 5, 6), which was also correlated with reduced uPA expression (Fig. 6j). It should be noted, however, that although the effect of double silencing on transwell cell invasion was dramatic showing significantly reduced invasion (Fig. 5), the effect on cell migration (Fig. 4) and tumor spheroid invasion (Fig. 6) was significantly reduced compared to RSU-1L depletion alone, but was not statistically significant compared to that of the control cells. This can be easily explained by the efficiency of silencing in each case. As expected depletion of *RSU-1L* was more efficient (Figs 1, 3) as it was shRNA-based stable infection whereas depletion of *RSU-1-X1* was less efficient as it was mediated by transient siRNA transfection. Therefore, rendering the effect of *RSU-1-X1* depletion less strong.

Moreover, results from the protein expression analysis in human BC samples verified our hypothesis that RSU-1L by itself is metastasis-promoting. That is why RSU-1L increases in metastatic human BC samples (Fig. 7a, compare lanes 4, 6 and 8 to 3, 5 and 7, as well as Fig. 7c). RSU-1-X1, in turn, is increased when *RSU-1L* is reduced and its increase likely leads to increased cell invasion, which also indicates that it is metastasis-promoting. However, RSU-1-X1 decreases in human metastatic samples (Fig. 7a, compare lanes 4, 6 and 8 to 3, 5 and 7 for RSU-1-X1, as well as Fig. 7c). Although there was some variation in expression among the 23 human BC samples tested by immunoblotting, as it is anticipated when dealing with patient samples, the results were overall consistent, as shown in the quantification analysis of the immunoblots (Fig. 7b,c). In general, high levels of RSU-1L are associated with low levels of RSU-1X1 and vice versa, which indicates a negative regulation between the two isoforms and it could suggest that the cell always needs the expression of one RSU-1 isoform, that is why it increases the expression of one when the other is lost as a compensation mechanism.

Moreover, even though it cannot be confirmed that cancer cells are not the only cell type that contributes to the expression of RSU-1 isoforms in the human samples, as other cells such as stromal cells, may also contribute at a smaller degree, these findings in human BC samples provide a clinical insight and corroborate our *in vitro* findings emphasizing the significance of RSU-1 in BC cell metastasis.

Regarding the interplay between RSU-1 and the PINCH-ILK-PARVA complex, we show that RSU-1X1 silencing in both cells lines results in downregulation of all three members of the PINCH-ILK-PARVA complex (Fig. 3b,c,g–i), suggesting a positive regulation of the complex by this isoform. As for the effect of *RSU-1L* silencing on PINCH-1 expression, it seems that stable depletion of *RSU-1L* in both cell lines reduces PINCH-1 expression regardless of the presence or absence of RSU-1X1 isoform (Fig. 3b,h). This appears to be opposite from the finding of our previous work in which *RSU-1* silencing promoted PINCH-1 expression^20^. A possible explanation could be related to the *RSU-1* expression levels. In the previous study^20^ both *RSU-1* isoforms where transiently silenced while in the present work we stably and efficiently eliminated only *RSU-1L*. Nevertheless, this is something that definitely needs to be investigated and clarified in more detail in the future.

With regard to ILK and PARVA, *RSU-1L* silencing reduces their expression in MCF-7 cells (Fig. 3b,g,i) but increases it in MDA-MB-231-LM2 cells (Fig. 3b,g,i). Although, this may initially sound contradictory, it, in fact, validates our hypothesis, since depletion of RSU-1L leads to upregulation of RSU-1X1 which in turn positively regulates ILK and PARVA thus promoting their expression. That is why double knock-down of both isoforms reverses this effect (Fig. 3b,g,i).

Based on our results, we propose a model, a summary of which is diagrammatically depicted in Fig. 7d. RSU-1 isoforms are tightly regulated in cancer, both having metastasis-promoting properties through activation of uPA and by inducing the expression of PINCH-ILK-PARVA complex. Furthermore, RSU-1L exhibits a suppressive effect on RSU-1-X1, which means that when RSU-1L is lost, RSU-1-X1 is upregulated to compensate for this loss, activating uPA and the PINCH-ILK-PARVA complex (Fig. 7d). Hence, in order for metastasis to be eliminated both isoforms need to be blocked.

The present work highlights the importance of *RSU*-1 isoforms in BC cell metastasis paving the way for the use of RSU-1 protein as a metastasis biomarker and/or potent therapeutic target to treat metastasis. Evidently, several questions need to be answered; for instance, it needs to be clarified whether the expression of the two isoforms correlates with tumor grade, which could render *RSU-1* a BC biomarker or whether *RSU-1* expression has similar significance in other types of cancer. Moreover, the molecular mechanism of RSU-1 action as well as its interactors needs to be investigated further. Finally, the effect of blocking the two isoforms in preclinical models of cancer would potentially offer insights into its use as a therapeutic target against BC metastasis. In fact, identification of blocking peptides or inhibitors of RSU-1 has the potential to lead to novel therapeutic approaches that will ultimately improve overall survival of cancer patients dealing directly with metastasis.

## Methods

### BC cell lines

BC cell line MCF-7 was purchased from ATCC, while MDA-MB-231-LM2 cells were kindly provided by Dr. Joan Massague, Memorial Sloan Kettering Cancer Center^24^. All cells were grown in Dulbecco’s Modified Eagle Medium supplemented with 10% Fetal Bovine Serum, 1% Glutamine and 1% Penicillin/Streptomycin, and incubated in a CO_2_-incubator at 37 °C^22^. All cell culture reagents were purchased from Invitrogen Life Technologies^22^.

### Plasmid construction and lentiviral-mediated shRNA transduction

To construct a lentiviral vector expressing small hairpin RNA (shRNAs) against *RSU-1L*, we used the AgeI/EcoRI-digested pLKO.1-puro vector ligated with 58 base pair-oligos^27^. The forward oligo sequence for *RSU-1L* shRNA construct is 5′CCGG**GCCAGAAGCAGGTATTCAAAG**CTCGAG**CTTTGAATACCTGCTTCTGGC**TTTTTG-3′ while the reverse oligo sequence is 5′AATTCAAAAA**GCCAGAAGCAGGTATTCAAAG**CTCGAG**CTTTGAATACCTGCTTCTGGC -**3′. Ligated plasmids were transformed into XL10 gold competent bacteria and selected on ampicillin-containing LB agar plates (100 μg/mL). Single colonies were grown overnight in LB broth and plasmids were isolated using a NucleoSpin_PlasmidQuickPure kit (Macherey–Nagel)^28^. The presence of each insert was tested by PCR (KAPA Biosystems) using pLKO.1 Forward primer: GGAATAGAAGAAGAAGGTGGA and *RSU-1L* Reverse primer: GCCAGAAGCAGGTATTCAAAG. The sequence of the construct was verified by DNA sequencing (Macrogen, Netherlands). Establishment of cells stably expressing *RSU-1L* shRNA or a vector containing scrambled shRNA (Scrambled shRNA-SshRNA control from Addgene) was performed by lentiviral-mediated transduction. Briefly, 293 T cells were co-transfected with 5 μg pLKO-SshRNA or pLKO-shRSU-1L plasmids, and specifically 1 μg pMD2.G (envelope plasmid) and 3 μg psPAX2 (packaging plasmid). After 48 h, cells were transduced with virus-containing medium in the presence of 10 μg/ml polybrene (Millipore), selected with 2 μg/ml puromycin (Acros organics), and pooled for further assays^27^.

### siRNA Transfection

BC cells (MCF-7 and MDA-MB-231-LM2) stably expressing either the scrambled SshRNA control or the RSU-1L-shRNA were transfected with 100 nM non-specific control (NSC) siRNA or siRNA against RSU-1X1 using the Lipofectamine 2000 reagent (Invitrogen Life Technologies). The siRNAs were obtained from Eurofins Genomics/VBC. As NSC, a sequence of 47% GC NSC was used while the sequence targeting RSU-1-X1 was 5′AGAACUAGCCUCUACGGCAUU 3′. Cells were harvested 48 h post-transfection and silencing efficiency was verified by western blot and real time PCR as specified in each experiment^22^.

### RNA isolation and Real Time PCR

Total RNA was extracted from BC cells using Trizol (Invitrogen) and purified using RNeasy mini kit (Qiagen)^22^. RNA concentration and purity was assessed using the IMPLEN nanophotometer. Pure RNA samples (with a A260/A280 ratio close to 2.00 but not lower than 1.80) were transcribed to cDNA using Superscript III Reverse Transcriptase (Invitrogen) following the company’s instructions^22^ and using 1 μg of RNA as starting material. The resulting cDNA was diluted 1:10 before being used for quantification of gene expression by real-time PCR using β-actin as reference gene. At least six replicates were run for each sample (three with the test primer and three with the β-actin primer) and each reaction had a total volume of 10 μl which included 1 μl of cDNA, 1 μl of the respective primer mixture (forward and reverse primer, 10 μM each), 3 μl of dH_2_O and 5 μl SYBR green reagent (Kapa SYBR Fast qPCR Master Mix-2x, Kapa Biosystems, Cat.#KR0389). Replicates were placed in a 96-well PCR plate (*4titude*) and the real time PCR reaction was performed in a CFX96 thermal cycler (BioRad). The real time PCR conditions were the same for all genes tested and included an initial step of 2 min incubation at 95 °C. This was followed by incubation at 95 °C for 2 sec, 60 °C for 20 sec and 72 °C for 30 sec, with 2–3 steps being repeated for 39 cycles. After the completion of the PCR, melting curves were monitored to ensure that one peak is detected and amplified. At least 3 independent experiments were performed. All primers used are shown in Supplementary Table 1. Quantification of relative gene expression was performed using the ΔΔCt method, where data were log transformed and analyzed using standard methodology^29^. Cells infected with S-shRNA SshRNA + Non Specific control (NSC) were used as calibrators, as specified in each experiment.

### Protein isolation and Western blot analysis

For protein expression analysis, standard immunoblotting protocol was followed, as described previously^22^. Total cell lysates were obtained from cell pellets using 1% sodium dodecyl sulfate (SDS) in RIPA buffer (20 mMTris/Cl pH 7.5, 150 mM NaCl, 0.5% NP-40, 1% TX-100, 0.25% sodium deoxycholated, 0.6–2 μg/ml aprotinin, 10 μM leupeptin, 1 μM pepstatin)^22^. Protein concentration was determined using the Bicinchoninic Acid (BCA) protein assay kit from Pierce (Cat.#23225) and absorbance was measured using an RT-2100C Rayto microplate reader at 562 nm. An amount of 20 μg of protein (that did not exceed 35 μl in volume) from each cell lysate was run on a 10–12% polyacrylamide gel (or 15% gel for human samples presented in Fig. 7) at 200 Volts for 40–60 min. Transfer to a PVDF membrane (Millipore) was achieved at 15Volts for 20 min using the BioRad Semi-dry transfer system (BioRad). Membrane was blocked in 5% non-fat milk in Tris-buffered saline-tween (TBST) buffer for 1 h and was then incubated with appropriate antibodies^30^. The exact antibodies used, as well as their working dilutions and incubations times are provided in Supplementary Table 2. Signal was visualized using chemiluminescent substrate from Pierce (SuperSignal West Femto Maximum Sensitivity Substrate, Cat.#34095) and Fuji Biomax light films (Cat.# RX1318) were developed manually using Carestream Kodak autoradiography GBX developer/replenisher (Cat.#P7042) and fixer/replenisher (Cat.#P7167) according to the company’s instructions. Films were then scanned using an HP Scanjet G4010 scanner and images were analyzed in Adobe Photoshop. Color was discarded and images were converted to grayscale. When needed, contrast was used on the entire image to enhance image clarity. No other image manipulation was performed^22^. Most blots were sequentially reprobed with suitable antibodies as indicated in each figure legend, without stripping. Each blot was reprobed with an antibody for loading (β-actin, GAPDH, or β-tubulin) and up to four (4) other relevant antibodies without stripping, as indicated. The full length blots are provided in the supplementary file of the manuscript (Supplementary Figs 1–5).

### Cell migration assay

Cell migration was assessed using the standard transwell migration assay. Equal number of cells per condition were suspended in 0.5 ml of plain DMEM and added to the upper chamber of the 8-μm pore diameter transwell motility chamber (2 × 10^4^ cells/chamber) while complete serum-containing medium (10% Fetal Bovine Serum) was added to the bottom of the transwell serving as chemoattractant. After incubation at 37 °C for 18 h, cells on the upper surface of the membrane were removed. The membranes were fixed with 4% paraformaldehyde (PFA) and the cells on the undersurface were stained with 0.1% Crystal Violet for 30 min. Finally, transwells were washed with distilled water and pictures were taken from five randomly selected microscopic fields using a Nikon Eclipse optical microscope equipped with a digital camera^31^. Cells in all 5 fields were counted. Experiments were run in duplicates and at least two independent experiments were performed.

### Cell invasion assay

Transwell invasion assay^32^ was analyzed using the BD biosciences tumor invasion system (with matrigel coated inserts). Briefly, cells were trypsinized and suspended in DMEM containing 1 mg/mL BSA at a concentration of 5 × 10^5^ cells/ml while complete serum-containing medium was placed at the bottom chamber. Cells were incubated at 37 °C for 18 h and non-invading cells that remained on the upside of the filter were removed. The invading cells were fixed with PFA, stained with crystal violet and photographed under a Nikon Eclipse optical microscope equipped with digital camera. The number of invading cells was quantified by counting cells from five randomly selected microscopic fields under the 10x objective^33^.

### Tumor spheroid invasion assay

Tumor spheroids were formed using the hanging drop technique^34–36^. A cell suspension of 2.5 × 10^4^ cells/ml was prepared and hanging drops were made using 20 μl from this suspension. Thus, hanging drops containing 500 cells each were placed on the inside of the cover of a culture dish and incubated at 37 ^o^C^22^. Formed spheroids were transferred into wells containing 1 mg/ml 3D collagen I gel (Collagen I high concentration solution, Corning Cat.# 354249) using sterile glass Pasteur pipettes and left to grow for the designated time (5 h for MDA-MB-231-LM2 cells and 18 h for MCF-7 cells). The transfer was performed three days later (for SshRNA or RSU-1L shRNA stable cells) (Fig. 2) or the next day (for cells transiently expressing NSC, or RSU-1-X1 siRNA) (Fig. 5). The diameter of the spheroids was monitored by a Nikon Eclipse optical microscope equipped with a digital camera. Pictures were taken immediately (time zero) and at the end of the designated time period. Cell invasion through the surrounding collagen was measured using the ImageJ software and the final spheroid size (average of the major and minor axis length) was compared to the initial size at time zero^22,37^. In experiments where cells were subjected to RSU-1-X1 siRNA-mediated silencing, siRNA transfection was performed 24 h prior to formation of hanging drops and invasion was monitored up until 72 h post-transfection. At least 8 spheroids were analyzed per condition and at least two independent experiments were performed^22^.

### BC tissue samples

BC tissue samples as well as normal adjacent tissue from the same patient were obtained from patients undergoing tumor excision surgery at the University Hospital of Larissa (UHL), Greece during the period of 2005 to 2007^20^. Normal adjacent tissue was verified to be normal by UHL pathologists. A total of 32 invasive BC samples and their normal counterparts were analyzed for *RSU-1* expression by Real Time PCR and western blotting and the data were published in a previous work^20^. Seventeen (17) samples were metastasis-free (*in situ*) and 15 were considered to be metastatic exhibiting metastasis to at least one lymph node (*metastatic*). In the present study, protein lysates kept from these samples were re-run on a 15% polyacrylamide gel, so that the smaller truncated isoform of RSU-1 (RSU-1X1) (~29KDa) is also distinguished on the gel, and analyzed by western blotting using anti-RSU-1 antibody. For the samples presented in this work, information on the patients’ age, family history of BC, height, weight, BMI, age of menarche and menopause, number of pregnancies, smoking habits, grade, infiltration of the blood vessels, nerves, skin or nipple, pathology evaluation, tumor diameter, ER, PR and Her2/neu status, and lymph node metastasis is summarized in Supplementary Table 3. All samples were obtained following verbal informed consent according to a protocol approved by the Institutional Review Board of the UHL (#12549) and in accordance with ethical guidelines of the 1975 Declaration of Helsinki^20^.

### Quantification of protein expression from western blots

The two isoforms of *RSU-1* (Fig. 7) were quantified compared to the GAPDH loading control using the National Institute of Health Image J software^22^. The mean intensity of protein bands from five (5) different immunoblots including 23 human BC samples was used for the quantification. A p value of 0.05 was considered as statistically significant.

### Bioinformatics analysis

*RSU-1L* (NM_012425.3) and *RSU-1-X1* (XM_005252552.4) gene sequences were initially run in NCBI Blast Nucleotide (Blastn). To create a visualization of BLAST results we used the Kablammo open source BLAST visualization tool^38^.

### Statistical analysis

Normality was tested using Statgraphics Plus ‘Test for normality’. All the data presented in this work (apart from the analysis of protein expression presented in Fig. 7) followed normal distribution and therefore t-test was used for comparison of means between two groups (e.g. SshRNA versus RSU-1L shRNA-treated cells). For the data that did not follow normal distribution (western blot data presented in Fig. 7b,c), the non-parametric Mann Whitney test was used. In all cases a *p-*value < 0.05 was considered statistically significant. We also followed a graded approach in data presentation where a difference < 0.05 was denoted by one asterisk (*), a difference < 0.01 was denoted by two asterisks (**) and a difference < 0.001 was denoted by three asterisks (***).

### Ethics approval and consent to participate

All human BC samples were obtained following verbal informed consent according to a protocol approved by the Institutional Review Board of the UHL (#12549) and in accordance with the ethical guidelines of the 1975 Declaration of Helsinki^20^.

## Supplementary information

Supplementary material

## Data Availability

All data generated and analyzed during this study are included in this published article (and its Supplementary Information Files). All data and protocols are readily available to the readers.
