# Supplementary material for: Depletion of Ras Suppressor-1 (RSU-1) promotes cell invasion of breast cancer cells through a compensatory upregulation of a truncated isoform
Source: Sci Rep. 2019 Jul 11;9:10050. doi: 10.1038/s41598-019-46575-0 (PMC6624310; doi:10.1038/s41598-019-46575-0)
Supplement: Supplementary file 1 — Supplementary material [file 41598_2019_46575_MOESM1_ESM.pdf]

Depletion of Ras Suppressor-1 (RSU-1) promotes cell invasion of breast cancer cells through a compensatory upregulation of a truncated isoform

Vasiliki Gkretsi[1,2]*, Maria Kalli[1], Christodoulos Efstathiades[3], Panagiotis Papageorgis[1,4], Vassilios Papanikolaou[5], Lefteris C. Zacharia[6], Aspasia Tsezou[5,7], Evangelos Athanassiou[8], Triantafyllos Stylianopoulos[1]*

[1]Cancer Biophysics Laboratory, Department of Mechanical and Manufacturing Engineering, University of Cyprus, Nicosia, Cyprus.

[2]Biomedical Sciences Program, Department of Life Sciences, School of Sciences, European University Cyprus, Nicosia, Cyprus

[3]The Center for Risk and Decision Sciences (CERIDES), Department of Computer Sciences, School of Sciences, European University Cyprus, Nicosia, Cyprus

[4]Biological Sciences Program, Department of Life Sciences, School of Sciences, European University Cyprus, Nicosia, Cyprus

[5]Laboratory of Cytogenetics and Molecular Genetics, Faculty of Medicine, University of Thessaly, Larissa, Greece

[6]Department of Life and Health Sciences, University of Nicosia, Cyprus

[7]Department of Biology, Faculty of Medicine, University of Thessaly, Larissa, Greece

[8]Departments of Surgery, University of Thessaly Medical School, Larissa, Greece

Supplementary Table 1: Nucleotide sequence of the primers used for mRNA expression analysis

Primer name	Sequence
**ILK**	Forward 5' GAC ATG ACT GCC CGA ATT AG 3'<br>Reverse 5' CTG AGC GTC TGT TTG TGT CT 3'
**PARVA**	Forward: 5’-CAATTCGACTCCCAGACCAT-3’<br>Reverse: 5’-TGGTCGAACAAGGTGTCAAA-3’
**PINCH-1**	Forward: 5′ CCG CTG AGA AGA TCG TGA AC 3′<br>Reverse: 5′ GGG CAA AGA GCA TCT GAA AG 3′
**RSU-1**	Forward: 5’ AGG CCA CAG AGC AAG GTC TA 3’<br>Reverse: 5’ CGT GCA ATC TCA AAA GCT CA 3’
**RSU-1L**	Forward: 5’ GCCAGAAGCAGGTATTCAAA 3’<br>Reverse: 5’ CCGTTCTGAGACATACAAATA 3’
**RSU-1-X1**	Forward: 5’ CCAACATGCTGGATGTCAACGG 3’<br>Reverse: 5’ CCCAGAACTAGCCTCTACGGC 3’
**UPA**	Forward: 5’GCTGCTGACCCACAGTGGAA-3’<br>Reverse: 5’AAAGTCATGCGGCCTTGGAG-3’
**β-actin**	Forward: 5′-CGAGCACAGAGCCTCGCCTTTGCC-3’<br>Reverse: 5′-TGTCGACGACGAGCGCGGCGATAT-3’

Supplementary Table 2: Dilution of antibodies used in immunoblotting

**Target protein**	**Company and catalogue number**	**Source**	**Working dilution and incubation time**
**β-actin**	Sigma-Aldrich Cat.# A2228	Mouse monoclonal antibody	-1:4000 in 5% w/v nonfat dry milk in 1X TBST*<br>-1h incubation at room temperature with gentle shaking
Glyceraldehyde-3-Phosphate Dehydrogenase (**GAPDH)**	Santa Cruz Biotechnology Cat.# sc-166574	Mouse monoclonal antibody	-1:100 in 5% w/v nonfat dry milk in 1X TBST<br>-1h incubation at room temperature with gentle shaking
**ILK**	Cell signaling Technology Cat.# 3862	Rabbit polyclonal antibody	-1:1000 in 5% w/v BSA** in 1X TBST<br>- Overnight incubation at 4°C with gentle shaking
**PARVA**	Cell signaling Technology Cat.#8190S	Rabbit polyclonal antibody	-1:2000 in 5% w/v BSA in 1X TBST<br>- Overnight incubation at 4°C with gentle shaking
**PINCH-1**	Cell signaling Technology Cat.# 11890S	Mouse monoclonal antibody	-1:500 in 5% w/v nonfat dry milk in 1X TBST<br>- Overnight incubation at 4°C with gentle shaking
**RSU-1**	Kindly provided by Dr. Mary Lou Cutler, Professor at the Uniformed Services University of the Health Sciences, Bethesda USA.	Rabbit polyclonal antibody	-1:2000 in 5% w/v nonfat dry milk in 1X TBST<br>- Overnight incubation at 4°C with gentle shaking.
**β-Tubulin**	Developmental Studies Hybridoma Bank Cat.#E7	Mouse monoclonal antibody	-0.5μg/ml working dilution from a 42μg/ml stock (1:84)

			in 5% w/v nonfat dry milk in 1X TBST<br><br>-1h incubation at room temperature with gentle shaking
**Secondary ***mIgGκBP-HRP-goat-anti-mouse antibody**	Santa Cruz Biotechnology<br><br>Cat.#sc-516102	Mouse IgGκ light chain binding protein conjugated to horseradish peroxidase	-1:5000 in in 5% w/v nonfat dry milk in 1X TBST<br><br>-1h incubation at room temperature with gentle shaking
**Secondary HRP-goat-anti-rabbit IgG antibody**	Santa Cruz Biotechnology<br><br>Cat.#sc-2004	Goat polyclonal antibody	-1:5000 in in 5% w/v nonfat dry milk in 1X TBST<br><br>-1h incubation at room temperature with gentle shaking

***TBST: Tris-buffered Saline buffer containing 0.1% Tween 20**
****BSA: bovine serum albumin**
*****HRP: horseradish peroxidase (HRP)**

Supplementary Table 3: Patient information on the samples used for immunoblotting

	Sample #4	Sample #9	Sample #10	Sample #11
**Age**	52	66	72	73
**Family history**	Mother with BC	No	No	No
**Height (cm)**	160	168	158	165
**Weight (Kg)**	88	77	104	80
**BMI**	34.37	27.28	41.66	29.38
**Smoking**	No	No	No	No
**Menarche**	11	14	14	13
**Menopause**	52	50	54	34
**Number of pregnancies**	0	2	3	2
**Tumor diameter**	3.5	2.5	3.3	6.3
**Pathology evaluation**	Invasive ductal carcinoma	Invasive ductal carcinoma	Invasive ductal carcinoma	Invasive ductal carcinoma
**Grade**	III	III	III	II
**Vascular infiltration**	No	No	Yes	Yes
**Perineural infiltration**	No	No	No	No
**Skin infiltration**	No	No	No	No
**Nipple infiltration**	No	No	No	No
**LN metastasis**	No	Yes (in sentinel LN)	Yes (7 out of 31 LNs)	Yes (10/18 LN along with the sentinel LN)
**ER score**	-	++	-	++
**PR score**	+++	-	+	+++
**Her2/neu score**	+++	+++	-	+++

BMI: body-mass index, LN: lymph node, ER: Estrogen receptor, PR: Progesterone receptor.

Full-length blots

The full length blots from which actual pieces are taken and shown in the paper's figures are shown below. Pieces cropped are indicated with dashed boxes.

Supplementary Figure 1: *full length blot for Figure 1b. Lysates from the two stable cells lines (MCF-7 and MDA-MB-231-LM2) lacking RSU-1L (or transduced with the control scrambled s-shRNA) were loaded on a 12% polyacrylamide gel. An empty lane followed and a duplicate of the last 2 samples was included (MDA-MB-231-LM2 with and without RSU-1L). The parts of the blot that were cropped to generate the actual Figure 1b of the paper are indicated with red dashed boxes and respective red arrows. The blot was probed with RSU-1 first and then reprobed with β-actin without stripping.*

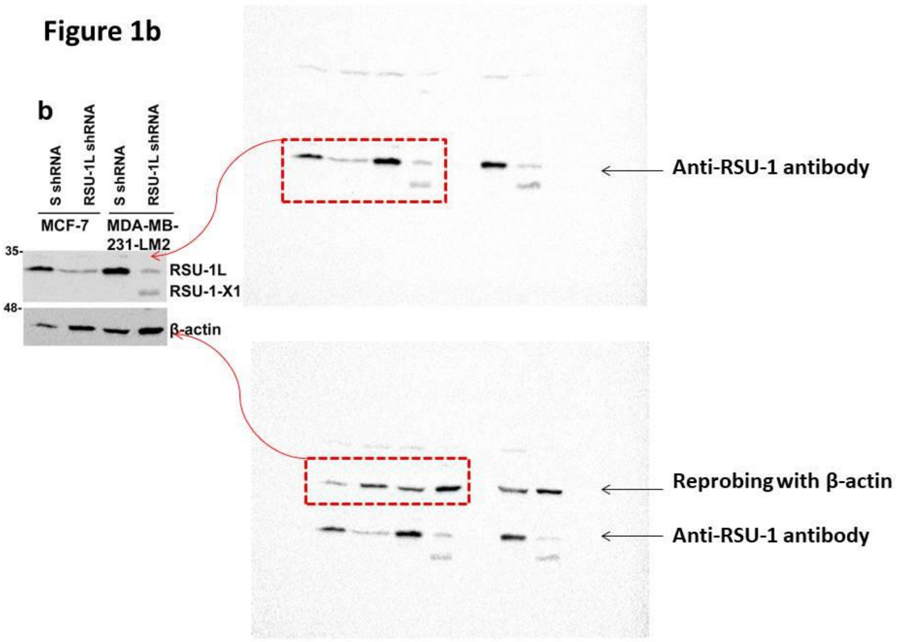

Supplementary Figure 2: full length blot for Figure 3a.
The blot was initially probed with ILK, then β-actin, then PINCH-1 and then RSU-1 antibody without stripping. The parts of the blot that were cropped to generate the actual Figure 3a of the revised paper are indicated with red dashed boxes and respective red arrows.

Figure 3a

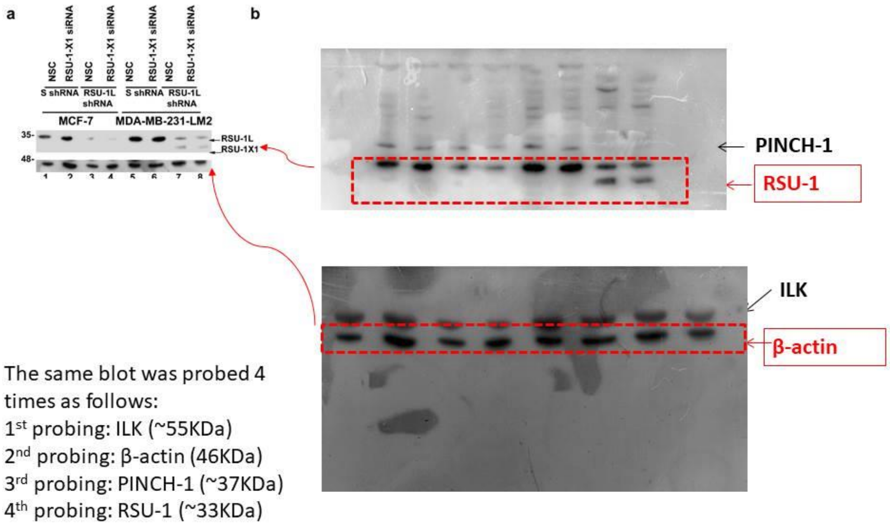

Supplementary Figure 3: full length blot for Figure 6a.
The blot was initially probed with rabbit anti-PINCH-1 antibody (~37KDa). The same blot was reprobed sequentially (without stripping) with mouse anti-ILK (~55KDa) and β-actin (~46KDa). The parts of the blots that were cropped to generate the actual Figure 3b are shown with red dashed boxes and arrows.

Figure 3b

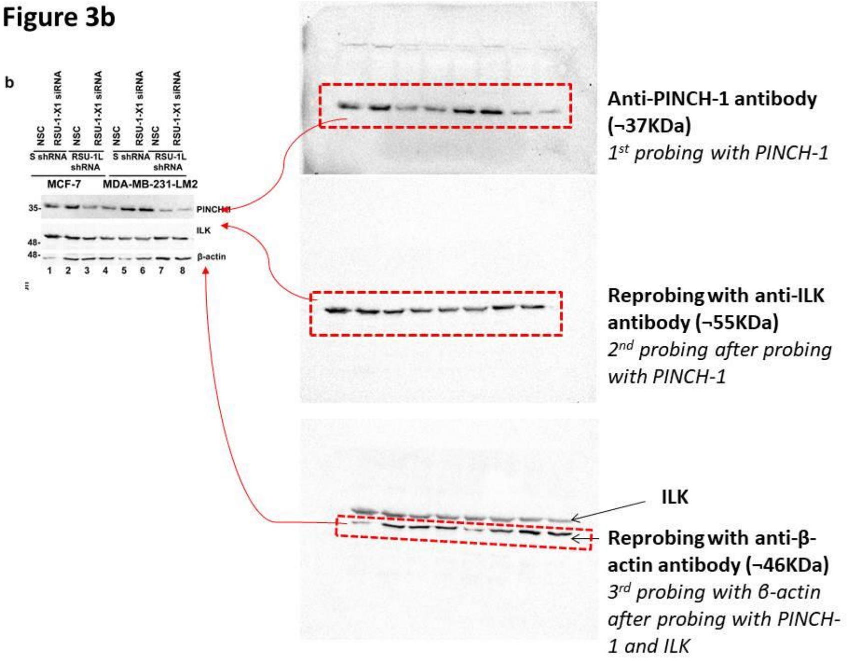

Supplementary Figure 4: full length blot for Figure 6b.
The immunoblot was initially probed with anti-PARVA antibody (~42 KDa) and reprobed with tubulin (~50-55KDa). The parts of the blots that were cropped to generate the actual Figure 6b are indicated with red dashed boxes and arrows.

Figure 3c

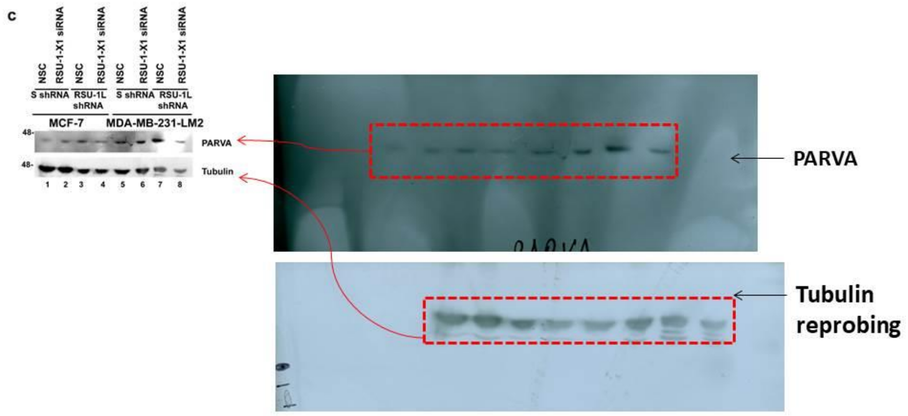

Supplementary Figure 5: full length blot for Figure 7a.
The particular Figure was generated from 2 different blots that included different human breast cancer samples. The blot was initially probed with anti-RSU-1 antibody and reprobed with anti-GAPDH antibody without stripping. The parts of the blots that were cropped to generate the actual Figure 7a are shown with dashed boxes and red arrows.

Figure 7a

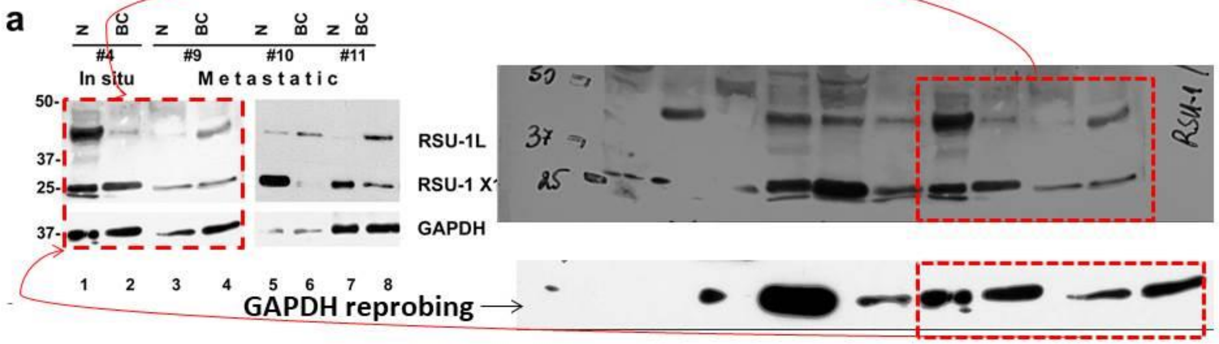

Figure 7a

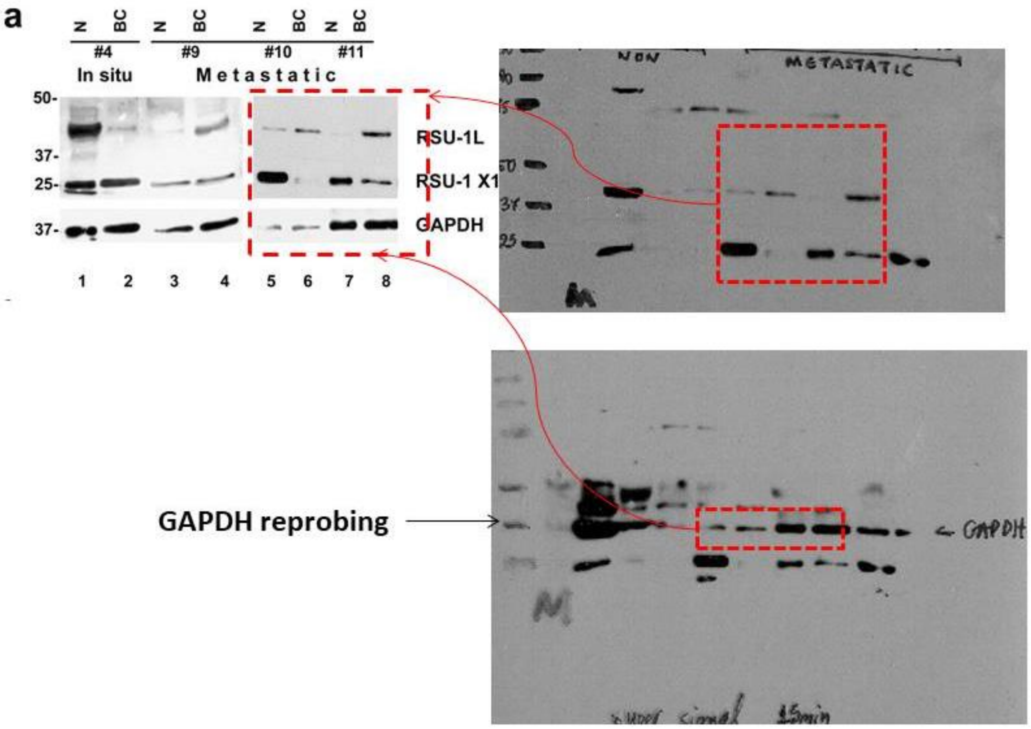

a
N
BC
N
BC
N
BC
N
BC
#4
#9
#10
#11
In situ
Metastatic
50-
37-
25-
37-
RSU-1L
RSU-1 X1
GAPDH
1
2
3
4
5
6
7
8
NON
METASTATIC
GAPDH reprobing
← GAPDH
M
